# Microstructure-Based Magneto-Mechanical Modeling of Magnetorheological Elastomer Composites: A Comparable Analysis of Dipole and Maxwell Methods

**DOI:** 10.3390/ma18051187

**Published:** 2025-03-06

**Authors:** Shengwei Feng, Lizhi Sun

**Affiliations:** Department of Civil and Environmental Engineering, University of California, Irvine, CA 92697-2175, USA; shengwf@uci.edu

**Keywords:** smart composites, magnetorheological elastomers, micromechanics, homogenization, magnetic interaction, particle distribution

## Abstract

Magnetorheological elastomers (MREs) are smart composite materials with tunable mechanical properties by ferromagnetic particle interactions. This study applied the microstructure-based dipole and Maxwell methods to evaluate the magneto-mechanical coupling and magnetostrictive responses of MREs, focusing on various particle distributions. The finite element modeling of representative volume elements with fixed volume fractions revealed that the straight chain microstructure exhibits the most significant magnetostrictive effect due to its low initial shear stiffness and significant magnetic force contributions. For particle separations exceeding three radii, the dipole and Maxwell methods yield consistent results for vertically or horizontally aligned particles. For particle separations greater than three radii, the dipole and Maxwell methods produce consistent results for vertically and horizontally aligned particles. However, discrepancies emerge for angled configurations and complex microstructures, with the largest deviation observed in the hexagonal particle distribution, where the two methods differ by approximately 27%. These findings highlight the importance of selecting appropriate modeling methods for optimizing MRE performance. Since anisotropic MREs with straight-chain alignments are the most widely used, our results confirm that the dipole method offers an efficient alternative to the Maxwell method for simulating these structures.

## 1. Introduction

Magnetostrictive or magnetorheological elastomers (MREs) are magnetic field-responsive smart composites whose primary constituents include ferromagnetic particles, an elastomeric matrix, and various performance-enhancing additives. The unique hallmark of MREs is their field-dependent mechanical behavior, which can be systematically tailored by modifying parameters such as the matrix composition, particle type and distribution, and the strength of the applied magnetic field. This tunability has enabled MREs to be adapted for diverse applications, including vibration-damping systems and adaptive robotics [1,2,3]. The magnetostrictive effect (MR effect) in MREs is intricately linked to the orientation of magnetic particles within the matrix. Traditionally, controlling this orientation during the curing process has been challenging due to difficulties in precisely manipulating the microstructural arrangement, often leading to variability in the properties of the final material. However, recent advancements in 3D printing technology have revolutionized the fabrication of MREs. Cutting-edge additive manufacturing techniques now enable precise placement of magnetic particles within the matrix, allowing for controlled anisotropic structures without the need for an external magnetic field during curing [4,5]. Given these developments, accurate modeling of magnetic interactions at the particle level is crucial for optimizing MRE design and performance.

Modeling the magneto-mechanical coupling in MREs has gained particular attention, as predictive models are required to capture the material responses under various operational conditions. These conditions encompass different magnetic field strengths, driving frequencies, strain amplitudes, and environmental factors [6,7,8,9]. Broadly, three modeling frameworks are employed to simulate the magnetostrictive behavior and magnetostrictive effect of MREs. The first is phenomenological approaches [10,11,12,13,14], which use the configurations of springs and dampers to replicate the dynamic stiffness and damping characteristics of MREs under various loading scenarios. The second framework, often referred to as the magnetoelastic or continuum mechanics method [15,16,17,18,19], treats MREs as continuous media, seamlessly incorporating the influence of ferromagnetic particles into the elastomeric matrix. This methodology provides deeper insights into the macroscopic properties of MREs through coupled equations of elasticity and magnetism. The third—and central to the focus of the present study—are microscale predictive models based on micromechanics [20,21,22,23,24], which analyze the microstructure of MREs by accounting for the distribution of particles and their magnetic interactions, thus providing a finer-grained view of how microparticle arrangements affect the macroscopic magneto-mechanical behavior of MREs.

Within micromechanics-based models, two main strategies are commonly employed to handle the magnetic interactions of particles. The first, often termed the “dipole method”, assumes that magnetizable microparticles behave as point-like dipoles [25]. This assumption enables the estimation of effective elastic moduli and magnetostrictive responses in the representative volume elements (RVEs) with different particle configurations. The second strategy is the “Maxwell method” [26], which leverages the Maxwell stress tensor to compute local magnetic and mechanical fields. Here, particles and the matrix are modeled distinctly without imposing restrictive assumptions about magnetization. Each approach offers advantages and limitations in terms of accuracy, computational cost, and applicability across different particle concentrations and magnetic field strengths, creating a need for systematic comparisons. While previous studies have compared the dipole and Maxwell methods, they were limited to their direct results without integrating them into the MRE modeling. This study not only examines the differences between these methods but also investigates how their results influence MRE simulations across various microstructures.

This study first uses a two-particle interaction model to compare the magnetic forces estimated by the dipole and Maxwell methods. Subsequently, microscopic Finite Element Method (FEM) simulations of RVEs, featuring both periodic and chain-like microstructures commonly found in MRE modeling, are conducted to evaluate the effective shear moduli of the composites. The magnetic effect, predicted by each method, is then incorporated into these RVEs as the body force in order to examine the resulting effective magnetostrictive effect with appropriate homogenization strategies. These findings offer a guideline for selecting an appropriate modeling framework under specific operating conditions, thereby facilitating more precise and reliable designs of MRE-based composite systems.

## 2. Methodologies

In the linear dipole approximation, each magnetic particle is treated as a point dipole whose magnetization responds linearly to the external field. This approach assumes particles are far from each other so that mutual induction and higher-order multipole interactions are negligible. For spherical particles, the magnetic moment of each particle is given by the following equation [25]:

(1) $$\vec{m}=\frac{4}{3}\pi r^{3}\vec{M},$$

where $\vec{M}=\chi \vec{H}$ is the magnetization, χ is the susceptibility, $\vec{H}$ is the magnetic field strength, and r is the radius of particles. In the case of a two-dimensional plane problem, considering two particles *i* and *j*, assuming that all the particles have the same magnetization intensity $\vec{m_{i}}=\vec{m_{j}}=\vec{m}$, the two components of the magnetic interaction force $\vec{F_{ij}}$ can be described using equations:

(2) $$F_{x}=\frac{4\pi \mu_{0}\chi^{2}H^{2}r^{6}}{3R^{4}}\left[\left(1-5\cos^{2}\theta \right)\sin\theta \right],$$

(3) $$F_{y}=\frac{4\pi \mu_{0}\chi^{2}H^{2}r^{6}}{3R^{4}}\left[\left(3-5\cos^{2}\theta \right)\cos\theta \right],$$

where $\mu_{0}=4\pi \times 10^{7}(H/m)$ is the vacuum permeability, θ is the angle, and R is the distance between the center of particles i and j.

The Maxwell method, in contrast, employs Maxwell’s equations to compute the magnetic potentials. The magnetic body force $F^{m}$ can be obtained using the following equation [26]:

(4) $$F^{m}=\oint_{\partial \Omega }\sigma^{M}\cdot \vec{n}dS,$$

where Ω is the boundary of the particle, $\vec{n}$ is the boundary normal, and $\sigma^{M}$ is the Maxwell stress tensor, which can be described by the following equation:

(5) $$\sigma_{ij}^{M}=\frac{1}{2}\left(H_{i}B_{j}+H_{j}B_{i}-B_{k}H_{k}\delta_{ij}\right),$$

where H is the magnetic field intensity vector, B is the magnetic flux density vector, and $\delta_{ij}$ is the Kronecker delta. The calculation of Maxwell forces is conducted numerically using the finite element method.

In the present modeling, both magnetic and mechanical effects are coupled, requiring consistent boundary conditions for each. For the mechanical response, we employ periodic boundary conditions (PBC) to ensure the continuity of displacements and tractions across opposite faces of the RVE. It is applied using the following relations:

(6) $$\left\{\begin{array}{c} u_{1}^{R}-u_{1}^{L}=\bar{\varepsilon_{xx}}a_{1},\\ u_{2}^{R}-u_{2}^{L}=\bar{\varepsilon_{yx}}a_{1}, \end{array}\right.$$

(7) $$\left\{\begin{array}{c} u_{1}^{T}-u_{1}^{B}=\bar{\varepsilon_{xy}}a_{2},\\ u_{2}^{T}-u_{2}^{B}=\bar{\varepsilon_{yy}}a_{2}, \end{array}\right.$$

where the displacements of the nodes on the left and right faces ($u^{L}$ and $u^{R}$), as well as those on the top and bottom faces ($u^{T}$ and $u^{B}$), are coupled to create the periodic boundary conditions. The corner node requires careful measurement and the application of coupling equations on both faces to which it is attached. Previous studies [24,27,28] have likewise employed periodic-type constraints for the magnetic boundary by specifying magnetic potentials at the RVE boundary and matching magnetic fluxes on opposing surfaces. In contrast, this work focuses on localized particle–particle interactions within the RVE and does not consider the influence of particles external to the RVE.

A common approach for homogenizing composite materials is the volume-averaging method, where the effective properties are determined by averaging the microscopic stress and strain fields over the volume of the RVE. In this study, the magnetic force is treated as a body force and applied in tandem with the displacement boundary conditions. According to [29,30], the effective stress in the presence of a body force can be expressed as follows:

(8) $$\bar{\sigma_{ij}}=\frac{1}{V_{\mu }}\int_{V_{\mu }}\left(\sigma_{ij}^{\mu }-f_{j}^{\mu }x_{i}\right)dV_{\mu },$$

where $\bar{\sigma_{ij}}$ is the effective stress, $V_{\mu }$ is the volume of the RVE, and $f_{j}^{\mu }$ is the magnetic body forces in the *j*-direction with a distance $x_{i\;}$ to the central axis. Alternatively, the boundary traction can also be used for homogenization using the equation:

(9) $$\bar{\sigma_{ij}}=\frac{1}{V_{\mu }}\int_{\Gamma_{\mu }}t_{j}^{\mu }x_{i}d\Gamma_{\mu },$$

where $\Gamma_{\mu }$ is the boundary of the RVE and $t_{j}^{\mu \;}$ is the reactive boundary traction in the *j*-direction with a distance $x_{i}$ to the central axis. In this study, under the assumption of linear deformations, both equations yield identical results. However, for nonlinear or time-dependent analyses, Equation (9) is recommended.

## 3. Results and Discussion

**Figure 1:**
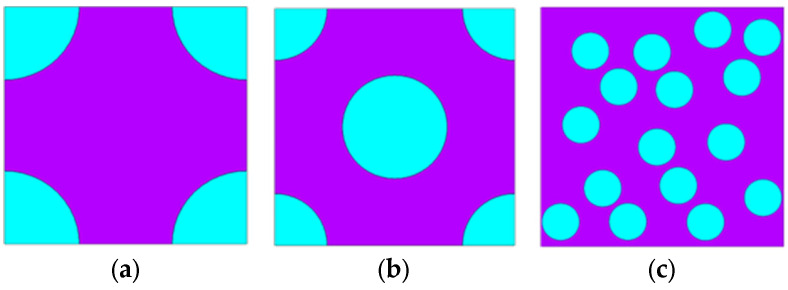
RVEs of MRE composites with (**a**) square, (**b**) hexagonal, and (**c**) random distributions of particles. The purple color represents the matrix, while the blue color represents the particles.

In the absence of an external magnetic field, the effective shear moduli of MREs were evaluated to examine the influence of particle distribution on the composite properties. Three particle distributions were considered, as illustrated in Figure 1: (a) square, (b) hexagonal, and (c) random. All particles were embedded in a square matrix, maintaining a constant volume fraction of particles at 30%, where the volume fraction of particles is defined as the ratio of the total volume of particles to the total volume of the RVE. Due to differences in particle arrangement, the total number of particles and their radii vary across the RVEs. For the random configuration, 100 RVEs with 16 particles of different particle locations were generated, each maintaining the same volume fraction. Small (0.1%) shear deformation was applied to each RVE in the finite element model to calculate the effective modulus, followed by homogenization of the resulting stress and strain fields.

**Figure 2:**
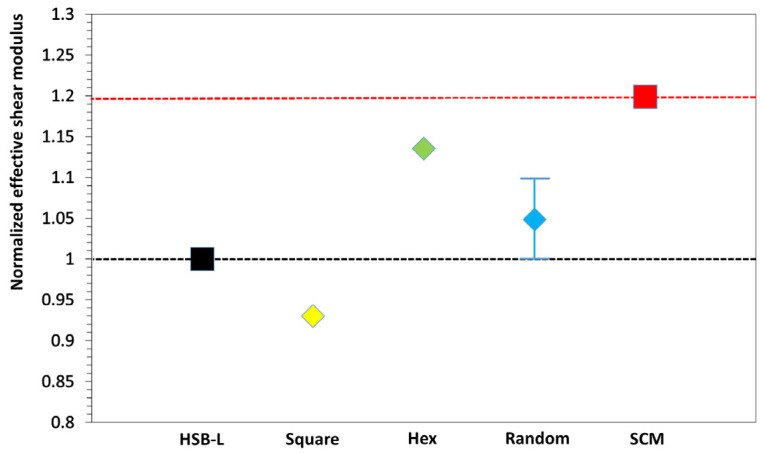
Effective shear modulus comparison for RVEs without magnetic field. The black dashed lines and red dashed lines indicate the upper and lower bound estimates given by HSB-L and SCM, respectively.

In addition to the FEM simulations, we include results from two widely used analytical methods: the Hashin–Shtrikman lower bound (HSB-L) [31], which provides a lower-bound estimate, and the Self-Consistent Method (SCM) [32,33], which offers an upper-bound prediction. These analytical bounds are represented by the dashed lines in Figure 2, indicating the range within which the effective modulus is expected to fall. The effective shear moduli are divided by the corresponding HSB-L values to obtain dimensionless ratios for comparison. For the random microstructure, the plotted range represents the variation in shear stiffness across a hundred different particle arrangements. The central marker indicates the average value, while the upper and lower limits represent the maximum and minimum values observed.

Due to the varying particle distributions, the effective shear modulus of random microstructures exhibits a variation of approximately 10%. However, these variations remain within the range predicted by the HSB-L and SCM estimates, indicating stable mechanical performances across different random configurations. In contrast, the two periodic microstructures demonstrate significant differences in behavior. The hexagonal distribution yields values close to the upper limit of the random distribution while still being below the upper bound provided by SCM. On the other hand, the square distribution shows a relatively low shear modulus, even lower than the HSB-L estimate. This suggests that due to its particle alignment, the square distribution behaves more like a layered material rather than an isotropic material, resulting in a reduced shear modulus. Since these particle distributions are frequently employed in MRE modeling, this finding highlights the need for caution, particularly when considering the magnetostrictive effect. RVEs with square particle patterns may exhibit a larger stiffness increase due to their initially lower shear modulus, which could lead to an overestimation of the magnetostrictive effect.

**Figure 3:**
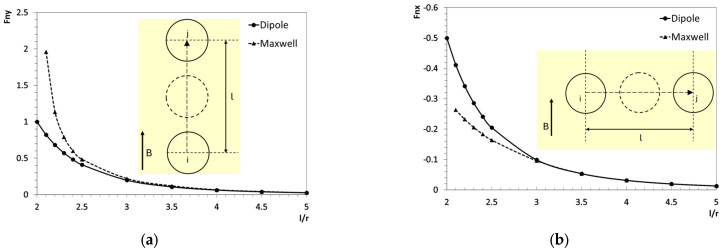
Magnetic interaction forces between two particles with (**a**) vertical and (**b**) horizontal alignment. The forces are calculated based on particle *i*, where positive values indicate attraction and negative values indicate repulsion.

The dipole and Maxwell methods are compared using a simplified two-particle system to evaluate their effectiveness under controlled conditions. A uniform magnetic field is applied vertically outward. Calculations using the dipole method were performed in MATLAB version R2023a (The MathWorks, Inc., Natick, MA, USA) using Equations (2) and (3). In contrast, the Maxwell method calculations were conducted in ANSYS Maxwell version 2021 R1 (ANSYS Inc., Canonsburg, PA, USA), employing appropriate mesh and boundary conditions to generate the uniform magnetic field. As shown in Figure 3, the two particles are aligned vertically and horizontally, and the relative inter-particle distance, *l*/*r*, is varied from two to five, where *l* represents the vertical distance between the particle centers and r is the particle radius. The magnetic force is analyzed using a dimensionless value $F_{n}=F/(\frac{\mu_{0}{V\chi }^{2}H^{2}r^{3}}{R^{4}})$ to facilitate a comparison between the methods, where $F_{n}\;$($F_{nx}\;or$ $F_{ny})$ is the horizontal or vertical components. Further, $\frac{\mu_{0}{V\chi }^{2}H^{2}r^{3}}{R^{4}}$ is set to $2\times 10^{-7}$ N, representing the magnetic attraction force calculated by the dipole method between two vertically aligned particles at a center-to-center distance of 2*r*. The positive values represent an attractive force between the particles, whereas negative values indicate repulsion. When the particles are positioned close to each other, near *l*/*r* = 2, the FEM simulation requires a significantly finer mesh to achieve convergence. To address this, the starting point for the Maxwell method simulations is set at *l*/*r* = 2.1.

Figure 3a illustrates the magnetic forces between vertically aligned particles, while Figure 3b presents the forces for horizontally aligned particles. A noticeable discrepancy is observed between the Maxwell and dipole methods when the inter-particle distance *l* ranges from 2*r* to 3*r*. In this range, the Maxwell method predicts significantly stronger attractive forces for vertically aligned particles compared to the dipole method, particularly as *l* approaches 2*r*. Conversely, for horizontally aligned particles, the Maxwell method estimates weaker repulsive forces than the dipole method. These differences gradually diminish as the distance increases, and by *l* = 3*r*, the results from both methods become nearly identical.

This discrepancy is more pronounced in Figure 3a, where particles are vertically aligned, highlighting a key consideration for real-world applications. In anisotropic MREs with chain alignments, where the vertical particle distance is generally smaller than the horizontal distance due to chain formation, this issue becomes even more significant. Therefore, caution is necessary when applying the dipole method in such cases, as it may not reliably capture nonlinear effects and higher-order interactions in close-range configurations. It is also important to note that the dipole method used in this study is based on the linear interaction dipole model. While more advanced dipole models accounting for multipole effects and nonlinear magnetization have been developed in previous studies [34,35], their implementation is beyond the scope of this work.

**Figure 4:**
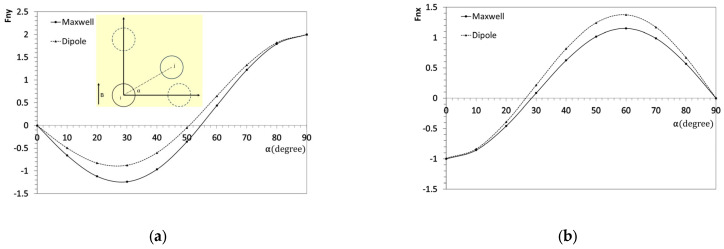
The x and y components of magnetic interaction forces between two particles with different angles (**a**) $F_{ny}$ and (**b**) $F_{nx}$.

Although the dipole method provides reasonable accuracy for *l* = 3*r*, our analysis thus far has only considered perfectly aligned particles, as seen in the square distribution. To address this limitation, we conducted additional simulations to account for particles with varying angular orientations. In this case, the distance between particle centers is fixed at *l* = 3*r*, and one particle is rotated around the other. The magnetic force components, $F_{nx}$ and $F_{ny}$, were calculated at 10° intervals from 0° to 90°, as shown in Figure 4.

The results reveal notable discrepancies in the rotation model. Specifically, the dipole method predicts higher absolute magnetic forces in the y-direction while underestimating forces in the x-direction. These trends align with the observations in Figure 3. The most significant divergence in the x-direction occurs at approximately $\alpha =60^{{}^{\circ}}$, where the dipole method overestimates the force by around 14%. Conversely, the most significant discrepancy in the y-direction is observed at $\alpha =30^{{}^{\circ}}$, with a 29% underestimation. The primary reason for these discrepancies lies in the assumptions of the dipole method. This approach assumes that the magnetic moment within all particles is uniform and aligned with the external vertical magnetic field. Additionally, it models particles as idealized dipoles, neglecting shape effects associated with their circular geometry. These simplifications lead to deviations, particularly in angular-dependent interactions where local field distortions and higher-order effects become significant. As a result, the dipole method may not fully capture the complex magnetization distribution present in real particle assemblies, necessitating caution when applying it to rotational analyses.

**Figure 5:**
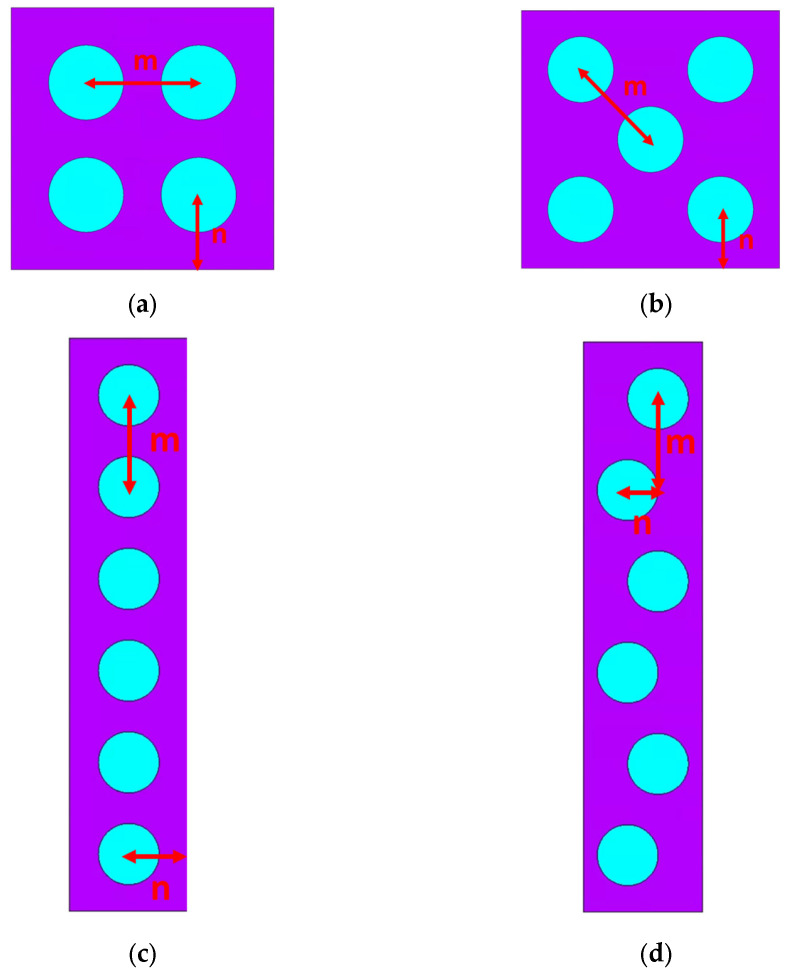
RVEs with various microstructures: (**a**) square, (**b**) hexagonal, (**c**) straight chain, and (**d**) wavy chain. The purple color represents the matrix, while the blue color represents the particles.

To further investigate this angular dependence, we propose four RVEs with different particle distributions: (a) square, (b) hexagonal, (c) straight chain, and (d) wavy chain, as illustrated in Figure 5. Maintaining a minimum inter-particle distance of 3*r* while controlling the volume fraction is challenging for the random microstructure. Additionally, the results for random microstructures exhibit significant variability, making them unsuitable for meaningful comparisons. Therefore, random microstructures were not included in this section. All configurations maintain the same volume fraction of 25.6%. The geometry of each RVE is adjusted to ensure a minimum inter-particle distance of *l* = 3*r*, with detailed particle distances provided in Table 1 using parameters m and n. Considering a linear elastic small deformation scenario, an elastic modulus of 200 GPa and a Poisson’s ratio of 0.2 is assigned to the particles. At the same time, the matrix is modeled with an elastic modulus of 0.2 MPa and a Poisson’s ratio of 0.49, reflecting its near-incompressibility. The relative permeability of chosen to be 4000 for the particle and 1 for the matrix.

In Table 2, we present the results of a mesh convergence and error analysis performed using the hexagonal distributed RVE model. Various mesh densities, from coarse to very fine, were tested under a 1% shear strain and applied magnetic force. The effective shear modulus was calculated for each mesh, with the finest mesh (30,302 elements) serving as the reference. Once the mesh surpasses roughly 3000 elements, the error in the effective shear modulus remains below 1%. To balance computational efficiency and accuracy, we selected a mesh size of 5547 elements (corresponding to a maximum element size of 0.2 µm) for all subsequent simulations. Because the other particle distributions share the same volume fraction and have similar geometric arrangements of particles and matrix, this mesh configuration is expected to provide similarly accurate results for those cases as well.

**Figure 6:**
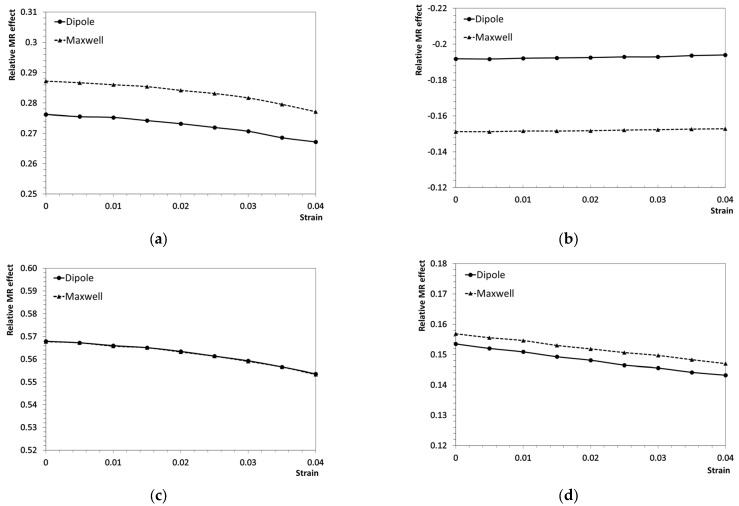
Comparison of the relative magnetostrictive effect between the dipole method and the Maxwell method for different particle distributions: (**a**) square, (**b**) hexagonal, (**c**) straight chain, and (**d**) wavy chain.

Assuming that the mechanical deformation of rigid particles due to magnetic forces is neglected, we investigate and compare the performance of the dipole and Maxwell methods under different conditions by first taking pairwise magnetic interaction forces from the two-particle model using both methods. In the RVE, each particle experiences the cumulative effect of interaction with all other particles such that the total magnetic force on the *i*-th particle is given by $F_{i}=\sum_{j\neq i}^{N}F_{ij}$, where $F_{ij}$ is the force on particle *i* due to particle *j* and N is the total number of particles. These magnetic body forces are applied within a finite element framework, coupled with a 4% macroscopic shear deformation introduced in eight increments of 0.5% each. Every increment is treated as a distinct boundary condition scenario, reflecting the evolving deformation state of the RVE. The effective shear modulus is then calculated via a unit-increment approach, where a small shear strain (0.1%) is imposed on the RVE for each boundary condition. The shear stress is evaluated both for the original, undeformed RVE and the RVE under the imposed strain, with Equation (9) used to sum the tractions on all boundary nodes. By dividing the difference in shear stress between these two configurations by the unit strain, we obtain the effective shear modulus. This procedure is repeated at each deformation level, allowing us to systematically assess how magnetic forces influence the RVE’s mechanical response under incremental shear loading. The relative MR effect is then quantified as $(G_{1}-G_{0})/G_{0}$, where $G_{0}$ is the effective shear modulus of the MRE composites without a magnetic field and $G_{1}$ is the field-dependent effective shear modulus with the applied magnetic field. The results are presented in Figure 6.

According to the results, the straight chain structure exhibits the highest MR effect, surpassing that of the square distribution. Meanwhile, the wavy chain configuration shows a lower positive MR effect than the square distribution but remains higher than the hexagonal distribution, where a negative MR effect is observed. This aligns with previous experimental findings, which indicate that anisotropic MREs tend to be more sensitive to applied magnetic fields and exhibit a larger MR effect compared to isotropic MREs [36,37]. Despite some differences in magnitude, both the dipole and Maxwell methods exhibit a similar trend in the MR effect with increasing strain amplitude. In Figure 6a,c,d, the positive MR effect decreases with shear strain, while in Figure 6b, the absolute negative MR effect increases with shear strain. This behavior can be attributed to the fact that the MR effect is primarily governed by the increment of the x-component of the magnetic interaction force that develops during deformation. Both particle distance and angular orientation influence this force. As strain increases, the inter-particle distance and angle change based on the specific particle arrangement, affecting the MR effect differently for each microstructure. This finding is consistent with previous experimental observations [8], where the MR effect was shown to vary nonlinearly with shear strain.

The straight chain microstructure in Figure 6c exhibits the most significant positive relative magnetostrictive, which is attributed to its vertical particle alignment and results in a relatively low initial shear stiffness and a large shear modulus increment under magnetic forces. Furthermore, the dipole and Maxwell methods yield identical results for this configuration, consistent with the findings in Figure 4, where both methods predict the same magnetic force when the particles are nearly vertically aligned. For the hexagonal distribution in Figure 6b, a negative relative magnetostrictive effect is observed. This occurs because the magnetic attraction transitions to repulsion at certain angles between particles, negatively contributing to the effective shear modulus. In this case, a notable difference is evident between the dipole and Maxwell methods, with the Maxwell method showing a more minor negative magnetostrictive effect than the dipole method. This can be explained using Equation (8), in which $F_{nx}$ governs the result obtained by homogenization. Referring to Figure 4b, the Maxwell method predicts a lower magnitude of $F_{nx}$, particularly in the middle range of angles. This effect is more pronounced when the nearest particle is at a 45-degree angle, as seen in Figure 6b. In Figure 6a, although the particles follow a square configuration, differences between the Maxwell and dipole methods still emerge. This is because diagonal particles, despite having a weaker magnetic interaction due to their greater separation, still contribute to the overall effect. These interactions create a negative magnetostrictive effect similar to that observed in Figure 6b. For the wavy chain microstructure in Figure 6d, the behavior is similar to that of the straight chain in Figure 6c. Since there is no lateral interaction from other chains, the overall behavior resembles that of a two-particle model. As expected, small differences between the dipole and Maxwell methods are still observed, but these differences are insignificant. The magnetostrictive effect is small but positive, which can be attributed to the specific particle arrangements in the wavy chain. A summary of the relative MR effects and the error between the two methods is presented in Table 3.

The wavy chain microstructure presents a unique case due to its asymmetric particle distribution relative to the central y-axis. When magnetic forces are applied, even in the absence of shear deformation, an initial traction force arises on the boundary solely from the magnetic forces. However, since the results are evaluated using the increment method, this initial traction force does not influence the findings. Another consideration is the torque generated by the interaction between the magnetic moments of the particles and the applied magnetic field. In the dipole method, all magnetic moments are assumed to be initially aligned with the external field in the positive y-direction. Because the applied shear deformation is small, the resulting torque due to particle rotation is negligible and can be safely ignored. For the Maxwell method, the magnetic moments of the particles are not perfectly aligned initially, leading to an initial torque. However, the change in torque caused by the small affine deformation remains minimal compared to the effect of the magnetic interaction forces. As observed in Figure 6, the relative magnetostrictive effect is primarily governed by the x-component of the magnetic forces, making the influence of torque insignificant in this context.

## 4. Conclusions

This study evaluated the mechanical and magnetostrictive behavior of MREs using the dipole and Maxwell methods, focusing on how the particle distribution influences their effective properties. While the Maxwell method generally provides more precise results, its computational complexity and mesh requirements can make it difficult to incorporate with other nonlinear effects. Consequently, we sought to identify under which conditions the dipole method can still achieve acceptable accuracy, balancing computational efficiency and modeling detail. Although previous studies have compared these two methods, they primarily focused on direct outcomes rather than integrating them into MRE modeling. This work not only examines the discrepancies between the Maxwell and dipole methods but also investigates how these differences influence MRE simulations across various microstructures.

The results indicate that, for a fixed volume fraction, the straight-chain microstructure exhibits the largest relative magnetostrictive effect, largely attributable to its lower initial shear stiffness and greater shear modulus increase under magnetic interactions. When the particle separation exceeds 3*r*, both the dipole and Maxwell methods produce consistent outcomes for vertically and horizontally aligned particles. However, discrepancies arise in angled configurations where the Maxwell method accounts for nonlinear and multipole effects that the dipole method neglects. Further analysis suggests that both approaches predict similar behavior for square and straight-chain distributions, whereas hexagonal distributions show significant deviations due to complex angular interactions, rendering the dipole method less reliable. For wavy-chain microstructures, the accuracy of both methods depends heavily on particle angles, necessitating careful evaluation of each approach.

Based on these findings, we recommend that users select their modeling method according to accuracy requirements and microstructural configuration. If only a general trend prediction is needed, both methods suffice, offering similar overall trends. However, for more precise modeling, attention must be paid to RVE selection and particle spacing. In particular, the dipole method remains reliable for square distributions when the minimum particle distance exceeds 3*r*. Although it can still be employed for wavy-chain structures, the potential for larger errors demands caution. In the case of hexagonal distributions, the dipole method is not recommended, as it fails to capture the shape effects and angular complexities that the Maxwell method more accurately represents.

Despite the dipole method’s limitations in reflecting short-range interactions or nuanced angular dependencies, it is computationally efficient and effectively captures overall magnetostrictive trends. This makes it particularly useful for large-scale simulations or preliminary investigations, whereas the Maxwell method is more appropriate for studies requiring higher accuracy and detailed nonlinear interactions. This work focused on linear elastic behavior, linear magnetization, and a specific set of particle arrangements. Future research should explore nonlinear deformations, multipole interactions, time-dependent effects, and external field influences to develop more comprehensive MRE models that can better mirror real-world applications.

## Figures and Tables

**Table 1:** Particle location inside RVEs.

Microstructure	m	n
Square	2*r*	1*r*
Hexagonal	2*r*	0.79*r*
Straight chain	2*r*	0.98*r*
Wavy chain	2*r*	1*r*

**Table 2:** Mesh convergence and error analysis.

Number of Elements	Shear Stress (Pa)	Error
1882	886.9	2.6%
2482	830.1	4%
3649	859.3	0.6% 0.01% 0.4%
5547	864.6	
9286	868.2	
18,812	859.3	0.6% 0%
30,302	864.5	

**Table 3:** Summary of different microstructures.

Microstructure	Relative MR Effect	Error
Square	29%	3.9%
Hexagonal	−15%	27%
Straight chain	56%	0.2%
Wavy chain	15%	2.5%

## Data Availability

The original contributions presented in this study are included in the article. Further inquiries can be directed to the corresponding author.

## References

[1] Ahamed R., Choi S.B., Ferdaus M.M. (2018). A state of art on magneto-rheological materials and their potential applications. J. Intell. Mater. Syst. Struct..

[2] Hooshiar A., Alkhalaf A., Dargahi J. (2020). Development and assessment of a stiffness display system for minimally invasive surgery based on smart magneto-rheological elastomers. Mater. Sci. Eng. C.

[3] Xu T., Zhang J., Salehizadeh M., Onaizah O., Diller E. (2019). Millimeter-scale flexible robots with programmable three-dimensional magnetization and motions. Sci. Robot..

[4] Krueger H., Vaezi M., Yang S. 3d printing of magnetorheological elastomers (mres) smart materials. Proceedings of the 1st International Conference on Progress in Additive Manufacturing (Pro-AM 2014).

[5] Bastola A.K., Hoang V.T., Li L. (2017). A novel hybrid magnetorheological elastomer developed by 3D printing. Mater. Des..

[6] Johnson N., Gordaninejad F., Wang X. (2018). Dynamic behavior of thick magnetorheological elastomers. J. Intell. Mater. Syst. Struct..

[7] Abramchuk S.S., Grishin D.A., Kramarenko E.Y., Stepanov G.V., Khokhlov A.R. (2006). Effect of a homogeneous magnetic field on the mechanical behavior of soft magnetic elastomers under compression. Polym. Sci. Ser. A.

[8] Gao W., Wang X. (2016). Steady shear characteristic and behavior of magneto-thermo-elasticity of isotropic MR elastomers. Smart Mater. Struct..

[9] Li W., Zhang X., Du H. (2012). Development and simulation evaluation of a magnetorheological elastomer isolator for seat vibration control. J. Intell. Mater. Syst. Struct..

[10] Li W.H., Zhou Y., Tian T.F. (2010). Viscoelastic properties of MR elastomers under harmonic loading. Rheol. Acta.

[11] Agirre-Olabide I., Berasategui J., Elejabarrieta M.J., Bou-Ali M.M. (2014). Characterization of the linear viscoelastic region of magnetorheological elastomers. J. Intell. Mater. Syst. Struct..

[12] Chen L., Jerrams S. (2011). A rheological model of the dynamic behavior of magnetorheological elastomers. J. Appl. Phys..

[13] Wen Y.K. (1976). Method for random vibration of hysteretic systems. J. Eng. Mech. Div..

[14] Yang J., Du H., Li W., Li Y., Li J., Sun S., Deng H.X. (2013). Experimental study and modeling of a novel magnetorheological elastomer isolator. Smart Mater. Struct..

[15] Kankanala S.V., Triantafyllidis N. (2004). On finitely strained magnetorheological elastomers. J. Mech. Phys. Solids.

[16] Liu H.T., Sun L.Z., Ju J.W. (2006). Elastoplastic modeling of progressive interfacial debonding for particle-reinforced metal-matrix composites. Acta Mech..

[17] Dorfmann A., Ogden R.W. (2006). Nonlinear electroelastic deformations. J. Elast..

[18] Bustamante R., Rajagopal K.R. (2015). Implicit constitutive relations for nonlinear magnetoelastic bodies. Proc. R. Soc. A Math. Phys. Eng. Sci..

[19] Bustamante R., Dorfmann A., Ogden R.W. (2011). Numerical solution of finite geometry boundary-value problems in nonlinear magnetoelasticity. Int. J. Solids Struct..

[20] Yin H.M., Sun L.Z. (2005). Magnetoelasticity of chain-structured ferromagnetic composites. Appl. Phys. Lett..

[21] Yin H.M., Sun L.Z. (2005). Elastic modelling of periodic composites with particle interactions. Philos. Mag. Lett..

[22] Danas K., Kankanala S.V., Triantafyllidis N. (2012). Experiments and modeling of iron-particle-filled magnetorheological elastomers. J. Mech. Phys. Solids.

[23] Han Y., Hong W., Faidley LA E. (2013). Field-stiffening effect of magneto-rheological elastomers. Int. J. Solids Struct..

[24] Mukherjee D., Bodelot L., Danas K. (2020). Microstructurally-guided explicit continuum models for isotropic magnetorheological elastomers with iron particles. Int. J. Non-Linear Mech..

[25] Jolly M.R., Carlson J.D., Muñoz B.C., Bullions T.A. (1996). The magnetoviscoelastic response of elastomer composites consisting of ferrous particles embedded in a polymer matrix. J. Intell. Mater. Syst. Struct..

[26] Landau L.D., Lifshitz E.M. (2013). Course of Theoretical Physics.

[27] Galipeau E., Rudykh S., Debotton G., Castañeda P.P. (2014). Magnetoactive elastomers with periodic and random microstructures. Int. J. Solids Struct..

[28] Metsch P., Kalina K.A., Brummund J., Kästner M. (2019). Two-and three-dimensional modeling approaches in magneto-mechanics: A quantitative comparison. Arch. Appl. Mech..

[29] De Souza Neto E.A., Blanco P.J., Sánchez P.J., Feijóo R.A. (2015). An RVE-based multiscale theory of solids with micro-scale inertia and body force effects. Mech. Mater..

[30] Blanco P.J., Sánchez P.J., de Souza Neto E.A., Feijóo R.A. (2016). Variational foundations and generalized unified theory of RVE-based multiscale models. Arch. Comput. Methods Eng..

[31] Hashin Z., Shtrikman S. (1963). A variational approach to the theory of the elastic behaviour of multiphase materials. J. Mech. Phys. Solids.

[32] Hill R. (1965). A self-consistent mechanics of composite materials. J. Mech. Phys. Solids.

[33] Budiansky B. (1965). On the elastic moduli of some heterogeneous materials. J. Mech. Phys. Solids.

[34] Keaveny E.E., Maxey M.R. (2008). Modeling the magnetic interactions between paramagnetic beads in magnetorheological fluids. J. Comput. Phys..

[35] Biller A.M., Stolbov O.V., Raikher Y.L. (2014). Modeling of particle interactions in magnetorheological elastomers. J. Appl. Phys..

[36] Böse H., Röder R. (2009). Magnetorheological elastomers with high variability of their mechanical properties. J. Phys. Conf. Ser..

[37] Kaleta J., Królewicz M., Lewandowski D. (2011). Magnetomechanical properties of anisotropic and isotropic magnetorheological composites with thermoplastic elastomer matrices. Smart Mater. Struct..

